# CEACAM6 expression and function in tumor biology: a comprehensive review

**DOI:** 10.1007/s12672-024-01053-6

**Published:** 2024-05-25

**Authors:** Dong Zhao, Fei Cai, Xuefei Liu, Tingting Li, Ershu Zhao, Xinlong Wang, Zhendong Zheng

**Affiliations:** 1 Department of Oncology, General Hospital of Northern Theater Command, Shenyang, China; 2 China Medical University, Shenyang, China

**Keywords:** CEACAM6, TME, Biomarkers, Therapeutic target, Vaccine therapy

## Abstract

Carcinoembryonic antigen-related cell adhesion molecule 6 (CEACAM6) is an immunoglobulin superfamily protein primarily expressed on epithelial surfaces and myeloid cells. It plays a significant role in cancer progression by inhibiting apoptosis, promoting drug resistance, and facilitating cancer cell invasion and metastasis. Overexpression of CEACAM6 has been observed in various cancers, including lung, breast, colorectal, and hepatocellular cancers, and is associated with poorer overall survival and disease-free survival. Its differential expression on tumor cell surfaces makes it a promising cancer marker. This review aims to provide a comprehensive summary of CEACAM6’s role in different cancer types, its involvement in signaling pathways, and recent advancements in CEACAM6-targeted treatments.

## Introduction

The CEA family was first described over 50 years ago [1, 2]. It was initially identified in primary and metastatic colorectal cancer, as dysregulation of CEACAM6 is the earliest molecular change observed in proliferative colon polyps and early adenomas [3, 4]. Subsequently, CEAs were found in various cancerous tissues and fetal tissues, and were found to have tumor-related immune functions in T cells, NK cells, and neutrophils [5, 6]. CEACAM6, also known as CD66c or NCA-90, is a non-specific cross-reactive glycoprotein antigen that shares some antigenic determinants with the family member CEACAM5 [7]. Both CEACAM5 and CEACAM6 are tumor-associated antigens that play important roles in cell adhesion and tumor cytochemical sensitivity. While CEACAM5 has received more attention, recent studies suggest that CEACAM6 may be a more promising target for antibody-based anti-metastasis and chemical sensitization therapy [8, 9]. This is due to the higher expression of CEACAM6 in cancer, as well as its correlation with prognostic indicators such as disease-free survival (DFS) and overall survival (OS) [10]. Among various cancer types, pancreatic cancer has been extensively studied in terms of CEACAM6 expression [11]. Differentially expressed proteins on the cell membrane surface, such as CEACAM6, are attractive therapeutic targets. As an important pathological biomarker in cancer, targeting CEACAM6 or the signaling pathway mediated by CEACAM6 has garnered significant attention in recent years.

## CEACAM6 and biomarker

Biomarkers are measurable indicators of exposure to normal and pathological processes or interventions. With the introduction of precision medicine, there is an increasing demand for biomarkers that can aid in early cancer detection, stratification, and personalized treatment. An ideal cancer biomarker should possess the following characteristics: (1) specific membrane expression in tumor cells, allowing for targeted therapy [12]; (2) differential expression between cancer cells, normal cells, and inflammatory cells; (3) differential expression associated with prognosis; and (4) easy detection with high sensitivity and specificity. Elevated levels of CEACAM6 are generally linked to a poor prognosis (Table 1). CEACAM6 has been found to be highly expressed in various common cancers, including colorectal cancer [13], pancreatic duct adenocarcinoma (PDAC) [14, 15], intrahepatic cholangiocarcinoma [16], gastric cancer (GC) [10], non-small cell lung cancer (NSCLC), and head and neck squamous cell adenocarcinoma (HNSCC) [17]. Detection of CEACAM6 in bodily fluids, such as blood or cerebrospinal fluid, is more clinically feasible than in tissues. For instance, in a study involving patients with PDAC, serum CEACAM6 expression was analyzed using ELISA and found to be high in patients with PDAC compared to those with chronic pancreatitis [14]. Additionally, CEACAM6 was also found to be highly expressed in the cerebrospinal fluid of patients with lung cancer meningioma metastasis (LUAD-LM) [18]. CEACAM6 has emerged as a promising biomarker.

**Table 1:** CEACAM6 is associated with poor prognosis

Cancer type	CEACAM6 overexpression	Clinical correlation	Reference
Pancreatic cancer	mRNA and protein	Postoperative survival, OS and DFS	[14, 23]
Non-small cell lung cancer	mRNA and protein	OS	[24]
Gastric cancer	mRNA and protein	OS and lung metastasis-free survival time	[6]
Lung adenocarcinoma	mRNA and protein	Metastasis	[18]
Colorectal cancer	mRNA and protein	OS and RFS	[13, 25]
Osteosarcoma	mRNA and protein	Lung metastasis status and OS	[26]
Cholangiocarcinoma	mRNA and protein	DFS and OS	[27, 28]
Bladder cancer	mRNA and protein	OS	[29]
Breast cancer	mRNA and protein	OS	[30, 31]

When CEACAM6 was used to distinguish benign from malignant tumors, it showed higher sensitivity (100%) compared to other biomarkers. In a recent study, gene expression analysis of global transcriptome data was used to identify a candidate IHC marker for Merkel cell carcinoma and small cell lung cancer (SCLC), the result showed that CEACAM6 is promising as a differentiator between two neuroendocrine tumor, and the combination of CEACAM6 and thyroid transcription factor can improve the sensitivity and specificity of SCLC detection [19]. Cutaneous metastases of pancreatic cancer are a rare finding [20, 21]. Tumors located in the body and tail of the pancreas were more likely to manifest skin metastases as an initial clinical manifestation (62.2%) than those in the head of the pancreas [22]. Skin metastases may be the first clinical manifestation of pancreatic cancer and represent a clear warning signal. In real-world clinical practice, many skin signs are often unrecognized due to the lack of accurate prevalence data. [21]. The study of CEACAM6 in the differentiation of Merkel cell carcinoma and SCLC provides ideas for the differentiation of primary skin malignancies and secondary malignancies, and we hope to have related studies in the future.

## Application in diagnosis

Molecular probes have emerged as valuable tools for the detection of tumors and neoplastic lesions, and they are being increasingly utilized in surgical navigation and fluorescent endoscopy. In 2016, a CEACAM6-mAb-Alexa Fluor 488 Probe Synthesis was developed to label living tissue in GC [32]. The development of a simple and accurate method for the detection of cancer during precancerous lesions is crucial for effective secondary prevention. In 2021, high expression of CEACAM6 was observed in precancerous lesions of GC, leading to the design of a new probe called CEACAM6 mAb-IRDye800CW, which can label gastric mucosal dysplasia obtained through endoscopic submucosal dissection (ESD) [33]. This probe has demonstrated success in labeling poor gastric mucosa and tracking metastasized tumors in vivo. However, further evaluation of the sensitivity and specificity of CEACAM6 probes requires a larger sample size.

Radiopharmaceutical theranostics has emerged as a promising approach in cancer intervention. Using the targeted antibody NY004, tumor labeling with [89Zr] and [177Lu] radionuclides has been achieved. [177Lu]-NY004 has shown suitability for pancreatic ductal adenocarcinoma (PDAC) therapy and single-photon emission computed tomography (SPECT) imaging. [89Zr]-NY004 positron emission tomography (PET) imaging has demonstrated effective tumor targeting and imaging efficiency, while [177Lu]-NY004 has exhibited significant therapeutic effects in a mouse PDAC model, providing valuable data for potential clinical applications [34].

## CEACAM6 and metastasis

Epithelial mesenchymal transition (EMT) is a process in which epithelial cells lose their polarity and intercellular adhesion, transforming into aggressive mesenchymal cells and acquiring migratory capabilities. Recent research has shown that EMT is not only involved in tumor genesis, migration, and metastasis, but also plays a role in drug resistance [35–37]. EMT is characterized by the loss of E-cadherin expression and the overexpression of mesenchymal cell markers [38, 39]. In various cancers such as PDAC, GC, colorectal cancer (CRC), cholangiocarcinoma (CCA), and osteosarcoma, CEACAM6 has been found to be involved in EMT regulation, tumor invasion, and metastasis [13, 26, 27, 40, 41].

In CCA, silencing CEACAM6 has been shown to significantly increase E-cadherin expression, decrease N-cadherin expression, downregulate TWIST transcription factors at both protein and mRNA levels, and reduce phosphorylation levels of molecules involved in the Src/PI3K/Akt signaling pathway [27]. This suggests that inhibition of CEACAM6 can inhibit the EMT process, thus achieving the purpose of inhibiting tumor metastasis and invasion. Similarly, in GC cells, CEACAM6 regulates EMT through the PI3K/Akt pathway, and treatment of CEACAM6 overexpressing cells with the PI3K inhibitor LY294002 can reverse the EMT process [41]. In pancreatic carcinomas, CEACAM6 is negatively correlated with EMT and promotes EMT through the ZEB1/ZEB2 pathway [40]. Additionally, CEACAM6 suppression can increase E-cadherin promoter activity in CRC [13].

Taken together, these findings suggest that CEACAM6 plays a crucial role in tumor progression by promoting EMT development.

## CEACAM6 and anoikis

Anoikis, a programmed cell death caused by insufficient or inappropriate adherent and attachment for cells to matrix [42], is a key mechanism that inhibits cell colonization and growth in a new matrix environment. The mechanism of drug resistance to anoikis is a prerequisite for tumor metastasis [43]. Studies have shown that overexpression of CEACAM6 induces anoikis resistance for the reason that CEACAM6 plays a critical role in ECM-cell adhesion process [44–46]. A majority of CEACAM6 on the surfaces of cancer cells can interact with other members of the CEA family on the surface of cancer cells in an interaction called cross-linking [47], which results in a significant increase in c-Src kinase activity and caveolin-1 phosphorylation. Inhibition of caveolin-1 expression almost completely eliminated the CEACAM6-induced increase in c-Src kinase activity and decrease in tyrl-527 phosphorylation. Another study also found that antibody mediated CEACAM6 increased its resistance to anoikis by 66% [48, 49]. Subsequently, in order to further explore the mechanism, it was found that the effect of CEACAM6 on the activation of Src-FAK signaling system increased in a dose-dependent manner. MAPK/signal-regulated kinase 1/2 (MEK1/2) and extracellular signal-regulated kinase (ERK) were phosphorylated, while no increase in EGFR phosphorylation was observed [47], on this basis, the silencing CEACAM6 gene partially restored anoikis resistance in PDAC [50]. Gene silencing can specifically target the gene of interest, but may also yield unwanted results and create safety concerns. Antibody 8F5 targeting CEACAM6 was designed in LUAD to identify the B domain of CEACAM6 and decreased cellular CEACAM6 expression, thereby markedly increasing anoikis sensitivity of the cell, the mechanism behind this via caspase activation especially caspase 9 and 10. Comparing with standard group, when combined with paclitaxel, anoikis activity increased from 37.19% to 49.2% on the basis of 18.51%, showing a synergistic effect [48]. CEACAM6 appears to play a large part in regulating anoikis via a Akt/c-src signaling pathway and may regulate gemcitabine resistance in IHCC and pancreatic cancer [28].

## Upstream regulator of CEACAM6

As mentioned previously, cisplatin (DDP) is currently a widely used platinum chemotherapy drug for cancer treatment. However, drug resistance remains a major challenge in cancer treatment. Recent studies have shown that circRNA and microRNA play important roles in various cancers [51–53]. Specifically, circRNAs can act as sponges for microRNAs (miRNAs), thereby affecting gene expression at the post-transcriptional level [52, 54]. In gastric cancer, it has been discovered that up-regulation of circ_0008035 can regulate the expression of CEACAM6 by sponging miR-1256 [55], thereby promoting the growth of gastric cancer. Targeted inhibition of circ_0008035 and miR-1256 has been shown to have the same effect on cells expressing Ki67 and MMP-2, as well as BAX-positive cells. This regulatory network of circ_0008035/miR-1256/CEACAM6 plays a crucial role in the development of GC, and targeted inhibition of this pathway may provide a novel approach for GC treatment.

Dysregulation of miRNAs has been associated with cancer progression and drug resistance. For instance, miR-146a and miR-26a are down-regulated in DDP-resistant LUAD cells and tissues [56]. These miRNAs directly target the 3-UTR of CEACAM6 and inhibit its expression, thereby increasing LUAD resistance to DDP. Similarly, in another study on LUAD, luciferase reporter gene assays confirmed that miR-29a inhibits the expression of CEACAM6 at the post-transcriptional level by binding to its 3-UTR, leading to the inhibition of growth, migration, and invasion of lung adenocarcinoma cells [57]. This mechanism of miRNA action on CEACAM6 has also been observed in pancreatic cancer, where miR-29a/b/c regulate CEACAM6 at the post-transcriptional level [40].

## CEACAM6 and signaling pathway

CEACAM6 and TGFβ/Smad pathway: TGF-β family members can regulate a series of cellular processes such as cell growth, differentiation and apoptosis, and the SMAD pathway is the most classical pathway of TGF-β. TGF-β binding to the type II receptor (TR II) enables TβRI to undergo heterotetramerization and be activated to phosphorylate the transcription factors SMAD2 and SMAD3, and the phosphorylated SMAD3 forms a complex with CO-Smad (SD4) to activate the expression of target genes. In GC, CEACAM6 has been confirmed as a target gene for TGF-β signal transduction [58]. The introduction of wild-type TGF-β II receptor into SUN638 cells with TGF-β II receptor mutation can restore TGF-β signal transduction, and the restoration of signal transduction induces the expression of CEACAM6. After that, SMAD2 or SMAD3 inhibitors were used to inhibit the down-pass of the signaling pathway, and it was found that this correlation effect was ineffective. CEACAM6 is a target of the TGF-β signaling pathway, mediated by SMAD3. This result is also consistent with another study in breast cancer, in which CEACAM6 is strongly associated with the development of invasive breast cancer in atypical ductal hyperplasia [31], and in the breast cancer molecular subtype HER2 overexpression (HER2-OE): The expression of CEACAM6 was highest in ER− , PR− , HER2 + , CK5/6 ± types, before which a functional synergy between HER2 and TGF-β had been demonstrated. HER2 and TGF-β can jointly regulate vascular endothelial growth factor VEGF, which is the target of transcription regulation of TGF-β-Smad. TGF-β or EGF on HER2-overexpressed breast cancer cells can induce SMAD3 phosphorylation, and CEACAM6 expression level is upregulated after treatment, but this correlation is not obvious in HER2-negative breast cancer cells.

**Fig. 1:**
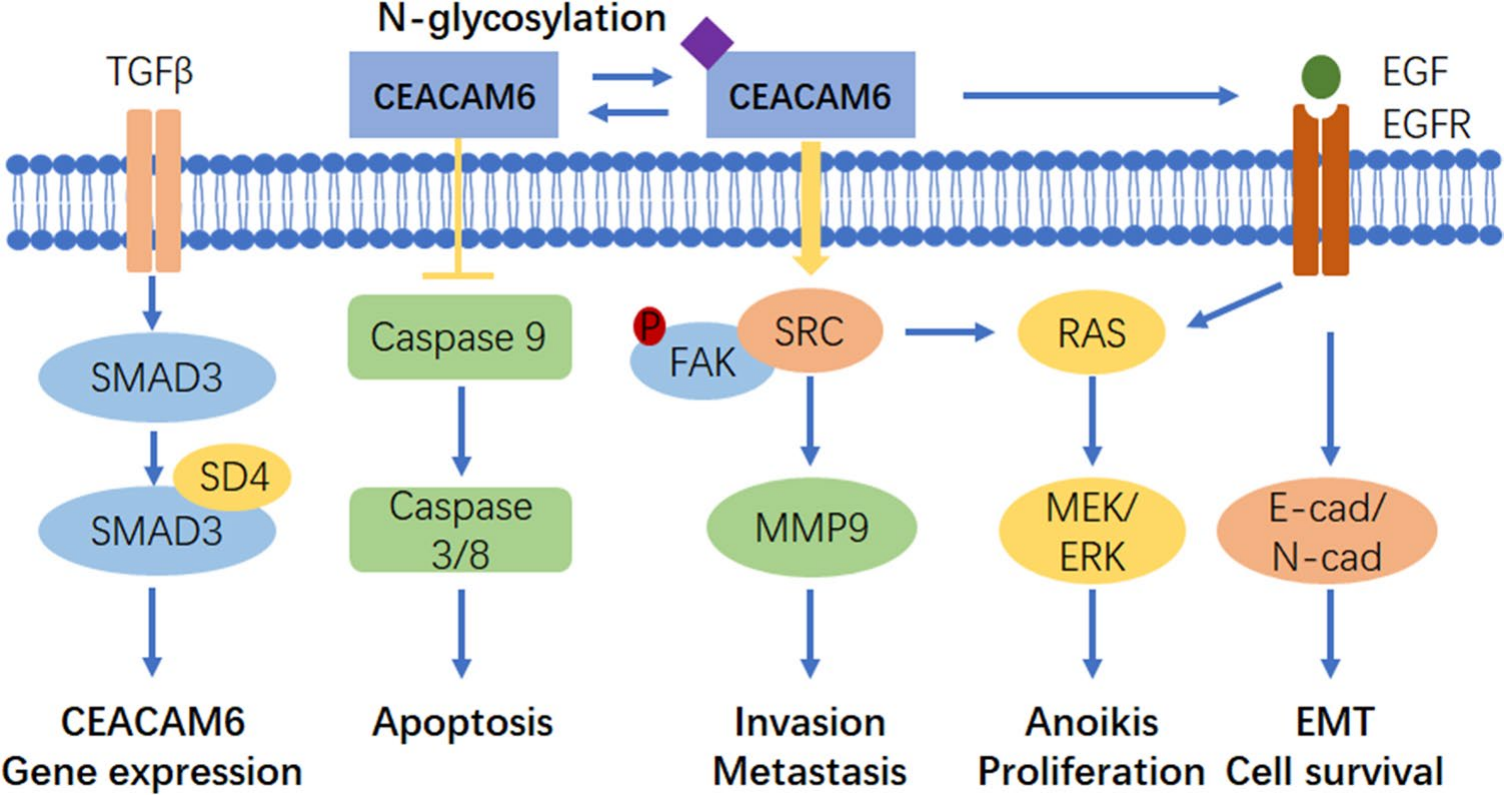
The mode of action of CEACAM6 in cancer

CEACAM6 and FAK pathway: Activation of focal adhesion kinase (FAK) occurs primarily through autophosphorylation in Tyr397 residues, and once activated, FAK phosphorylation is involved in a number of different downstream signaling pathways, such as FAK/PI3K/AKT. FAK ensures the transmission of survival signals through integrin-dependent adhesion and signaling. CEACAM6 plays an important role in the adhesion process of ECM cells, and overexpression of CEACAM6 can induce anoikis resistance. In breast cancer, CEACAM6 and CEACAM8 molecules are co-expressed to inhibit the invasion and proliferation of MCF-7 cells [59]. CEACAM6 cross-links to BXPC3 cells, resulting in ceavolin-1-dependent activation of Src, accompanying with increased phosphorylation of Src substrate FAK [11]. This mechanism may explain the mechanism by which CECAM6 is acquired in anoikis resistance. CEACAM6 siRNA silencing reversed anoikis resistance in MIA PACA-2 cells, and inhibition of CEACAM6 led to decreased anoikis resistance. Through the observation of the signaling system, it was found that the effect of CEACAM6 on the activation of Src-FAK signaling system was increased in a dose-dependent manner [47]. Another study also found that the resistance of antibody mediated CEACAM6 to anoikis was increased by 66%. Based on these, relevant antibodies were designed to silence the CEACAM6 gene and reverse resistance to anoikis in PDAC. However, gene silencing may bring safety issues. Antibody 8F5 targeting CEACAM6 was designed in lung adenocarcinoma to recognize the B domain of CEACAM6 [48], reduce the expression of CEACAM6 in cells, and thus significantly improve the anoikis sensitivity of cells through activation of caspase. Specifically, caspase 9 and 10. Compared with the standard group, anoikis activity increased from 37.19% to 49.2% on the basis of 18.51% when combined with paclitaxel, showing synergistic effect. The mode of action of CEACAM6 is shown in Fig. 1.

## Targeting CEACAM6 in *cancer* therapy

In addition to being a diagnostic marker, CEACAM6 is also an effective candidate therapeutic target. Designing targeted therapeutic drugs or vaccines is a common idea. Numerous studies have provided preclinical basis to support the feasibility of targeting CEACAM6 therapy, and both upstream and downstream factor-related inhibitors of the CEACAM6 pathway have potential applications [12, 60]. Gemcitabine is the standard treatment in PDAC, and intrinsic or acquired resistance can significantly reduce the effectiveness of chemotherapy [61]. SdAb, a single-domain antibody against CEACAM6 2A3, was designed to delay the proliferation, invasive and formation of tumor blood vessels of BXPC3 cells [62].

One notable characteristic of pancreatic ductal adenocarcinoma (PDAC) is the heightened activity of matrix metalloproteinase (MMP), particularly MMP-2 and MMP-9. Suppressing the secretion and activity of these MMPs can effectively impede the invasive capabilities of pancreatic cancer cells [62]. However, it is important to note that gemcitabine, a common chemotherapy drug used in PDAC treatment, does not possess the ability to inhibit the activity of MMP-9. In this context, Single-domain antibody (SdAb) emerges as a promising adjunct to gemcitabine, as it has been shown to effectively reduce the activity of MMP-9, complementing the treatment approach for PDAC. SdAb can reduce the activity of MMP-9 as a supplement of gemcitabine. In patients with NSCLC [63], monovalent SdAb, bivalent SdAb(Ab) and quadrivalent SdAb were used to inhibit the phosphorylation FAK domain Ty397 of the FAK/Src pathway regulated by CEACAM6, effectively inhibiting EMT-mediated migration and invasion, and the targeting ability of bivalent was superior to that of quadrivalent. Chemotherapy has always been the primary treatment option for most cancers, combination therapy, such as the combination of targeted inhibitors and chemotherapy agents, has become a trend [64]. In LUAD, CEACAM6 is associated with paclitaxel resistance. Anti-CEACAM6 mAb 8F5 has a significant synergistic effect when used in combination with the chemotherapy drug paclitaxel, showing 80% tumor growth inhibition in vitro xenograft models [48]. This is also the first demonstration that a monoclonal antibody against CEACAM6 can inhibit tumor growth in a tumor model.

Tumor immune microenvironment (TIME) and tumor antigen expression are key factors in the development of immunotherapy. In multiple myeloma, anti-CEACAM6 not only inhibited IFN-γ secretion of ex vivo isolated and resting, but also of polyclonally activated CD8 + T cells from multiple myeloma patients [65]. CEACAM6 is attached to the cell surface, and when CEACAM6 is overexpressed, it inhibits the phosphorylation of ZAP70 to inhibit T cell receptor-mediated T cell responses. Cis-or trans-dimerization between CEACAM family proteins is presumed to play an important role in the interaction of cell clusters composed of various cell types in TIME, where CEACAM1 is present on activated T cells [66], It is one of the few CEACAM receptors containing the intracellular immune receptor tyrosine-based inhibitory motivation (ITIM) domain for T cell signaling. Interaction between CEACAM6 on human solid cancer cells and CEACAM1 on activated tumor-reactive T cells suppresses the antitumor function of T cells, and a humanized anti-CEACAM6 antibody, BAY 1834942, blocks this interaction and restores the antitumor activity of T cells in CEACAM6-positive tumors [67]. The interaction and restores the antitumor activity of T cells in CEACAM6 and CEACAM1 is independent of the PD-1/PD-L1 axis, and BAY1834942 has a superimposed effect when used in combination with anti-PD-1/PD-L1 inhibitors. The Phase 1 of BAY1834942 in advanced solid has been completed (NCT03596372).

Small molecules targeting CEACAM6, such as siRNA, are also developed, which are often used to regulate endogenous gene expression, and most of them play an inhibitory role [68]. siRNA can bind to mRNA 3’UTR to silence genes and reduce their expression, which plays an inhibitory role. SiRNA delivery technology has also received widespread attention for its safety and good tolerability of lipid nanoparticle configuration in the first human clinical trial. Therefore, the vector is set to deliver CEACAM6-targeting SiRNA in a low PH-induced transmembrane structure (PHLIP) that can target multiple solid tumors. When combined with the chemotherapy drug cisplatin, the tumor inhibition effect of the included mice is obvious [69, 70]. In summary, all the results confirm that targeting CEACAM6 expression have tumor inhibitory effects, and the combination of targeted inhibitors and chemotherapy drugs is feasible.

## Vaccine therapy

4-1BB (CD137) and 4-1BBL are important T cell immune regulatory factors [71, 72]. Recombinant attenuated Salmonella carrying 4-1BBL gene can effectively enhance the T cells activities and inhibit the progression and metastasis of mouse colorectal cancer [73]. After constructing eukaryotic expression plasmid containing both CEACAM6 and 4-1BBL genes, attenuated Salmonella typhimurium carrying two genes was given to MC38 tumor model mice, and the size change of colorectal cancer model, the phenotype of tumor cells and the level of immune tissue infiltration of cancer tissue were observed. It was observed that the vaccine could effectively inhibit colorectal cancer [74–76]. Bivalent vaccine could increase the infiltration of CD3 T cells, CD8 T cells and NK cells, enhance T cell activity, decrease the infiltration of regulatory T cells, promote Th1 polarization, and inhibit Th2 and Th17 polarization [77]. Checkpoint inhibitors have been widely used in recent years, but only a small number of patients benefit from immunotherapy, for example in CRC, only patients with high microsatellite instability benefit, and this group accounts for less than 10% of CRC. At the same time, the presence of ITME is also a major obstacle to immunotherapy. CEACAM6-targeting immunotherapy has evolved from simple CEACAM6-depletion methods, such as Abs and small-molecule inhibitors, to more complex combination therapies, such as applying DNA vaccines in conjunction with chemotherapy. Injecting attenuated vaccine with PD1 inhibitor nebuliumab into mouse models showed that the combination therapy could enhance anti-tumor immune function and improve the therapeutic effect of colorectal cancer [76], which was further improved than that given alone.

CEACAM6 has become an attractive therapeutic target in many malignancies, and many antibodies are moving from preclinical studies to clinical trials. However, the application of antibodies or vaccines targeting CEACAM6 in clinical practice still faces many challenges. CEACAM6 can bind itself or CEACAM1, whether blocking CEACAM6 by antibodies alone is enough to restore the immunity of cancer patients remains to be discussed. In addition, current clinical trials tend to evaluate the additional efficacy benefit of anti-CEACAM6 antibodies in combination with established anticancer agents, but their safety and interaction with the immune microenvironment require more clinical data validation.

## Conclusion

In this comprehensive review, we delve into the multifaceted role of CEACAM6 within the tumor microenvironment, highlighting its functional significance in tumorigenesis. The review elucidates the diverse mechanisms regulating CEACAM6 expression and its consequent impact on cancer progression, encompassing both its promotive and suppressive roles in various cancer types. Moreover, we dissect the intricate signaling pathways influenced by CEACAM6, underscoring its potential as a biomarker for cancer diagnosis and prognosis. The latter part of the review is dedicated to exploring innovative therapeutic strategies targeting CEACAM6, evaluating their effectiveness and challenges. We assess the current landscape of targeted therapies, including monoclonal antibodies, small molecule inhibitors, and emerging approaches such as gene therapy, which are at the forefront of translating CEACAM6’s biological functions into clinical applications. This review aims to provide a thorough understanding of CEACAM6’s role in cancer, serving as a foundation for future research and development in cancer therapeutics.
